# A 21-Day School-Based Toothbrushing Intervention in Children Aged 6 to 9 Years in Indonesia and Nigeria: Protocol for a Two-Arm Superiority Randomized Controlled Trial

**DOI:** 10.2196/14156

**Published:** 2020-02-21

**Authors:** Paulo Melo, Sinead Malone, Arathi Rao, Charlotte Fine

**Affiliations:** 1 University of Porto Faculty of Dentistry, Institute of Public Health Epidemiology Research Unit Porto Portugal; 2 Unilever Oral Care Bebington Wirral United Kingdom; 3 Unilever Oral Care Blackfriars London United Kingdom; 4 FDI World Dental Federation Geneva-Cointrin Switzerland

**Keywords:** school children, oral health, OHIs, DMFT, school program, knowledge transfer, behavior change

## Abstract

**Background:**

The World Health Organization reports that dental cavities affect 60% to 90% of children globally. FDI World Dental Federation and Unilever Oral Care have developed public health programs to improve brushing habits over their 12-year partnership. The last of these (phase III) named Brush Day & Night aimed to educate children on brushing twice daily with a fluoride toothpaste and gave useful information for a new project, phase IV. The 21-day Brush Day & Night program is an intense education activity designed to establish the habit of brushing day and night with a fluoride toothpaste. The program involves daily brushing instruction and includes free toothpaste and toothbrushes.

**Objective:**

The main objective of the study is to evaluate the impact of a 21-day school program on children’s oral health. As a secondary objective, we aim to evaluate the impact on the knowledge, behavior, toothbrushing habits, and quality of life in school children aged 6 to 9 years after a 21-day school program and compare with baseline and a control group as measured by the self-reported questionnaires issued to children (in particular, the self-reported brushing frequency and positive responses on fluoridated toothpaste use). The enduring nature of the program will be determined by the inclusion of 8- and 24-week time points.

**Methods:**

The study is a 2-arm superiority randomized controlled trial. Clusters in this study are infant and junior schools in Indonesia and Nigeria. The study aims to recruit 20 schools with children aged 6 to 9 years in each country. At baseline, children in both intervention and control schools will answer a questionnaire and have their clinical oral health assessed using the Simplified Oral Hygiene Index (OHI) and Decayed Missing and Filled Teeth index. Children in the intervention schools will then take part in a structured 21-day Brush Day & Night intervention. Children in the control schools will be provided with free toothpaste and toothbrushes but will not receive the 21-day intervention. The questionnaires and OHI assessments are repeated after the 21-day program is completed and again 8 weeks later and 24 weeks later for all participating children. Parents/carers/guardians of all children will sign the informed consent and complete questionnaires on their own experience and attitudes toward oral health and toothbrushing routine at each of the four times points (baseline, 21 days, 8 weeks, and 24 weeks). The study will be conducted by the national dental associations of Indonesia and Nigeria and was approved by the ethics committees of both countries.

**Results:**

The study is ongoing. Recruitment of schools started in Indonesia in February 2018 and in Nigeria in April 2018 for the first part of the study, which concluded in Indonesia in September 2018 and in Nigeria in November 2018. The second part of the study (the second half of the schools) started in November 2018 in Indonesia and December 2018 in Nigeria.

**Conclusions:**

We expect to collect all the data during 2019 and publish findings from the study by March 2020.

**Trial Registration:**

ClinicalTrials.gov NCT04001296; https://tinyurl.com/selxraa

**International Registered Report Identifier (IRRID):**

DERR1-10.2196/14156

## Introduction

### Prior Work

The Brush Day & Night (BDN) program is the result of a 12-year partnership between FDI World Dental Federation (FDI) and Unilever. It is an intense education activity designed to establish the habit of brushing day and night with a fluoride toothpaste. The program involves daily brushing instruction and includes free toothpaste and toothbrushes (see Multimedia Appendix 1 for more information).

The program was designed based on the theories of behavior change. Behavior change interventions and the formation of health promoting habits is thoroughly reviewed by Lally et al [1] and specifically in the context of interventions to improve tooth brushing by Claessen et al [2]. This and other research was crystallized into the Unilever 5 levers of change model [3], which is the foundation for the design of this particular intervention. The first step of the model is to identify barriers, triggers, and motivators to adopting a new behavior. Those insights are considered in designing a new behavioral intervention in 5 steps:

- Make it understood
- Make it easy
- Make it desirable
- Make it rewarding
- Make it a habit

This is a 21-day intervention, a duration chosen to reflect past research [4] that repetition of between 12 and 15 times is necessary to make a habit change, and 21 calendar days is 15 school days. The choice of schools within which to implement the program was influenced by the work of Pine et al [5] that states schools can be an optimum place for behavior change interventions where both parents and teachers may be involved in the intervention. The duration of 21 days was also chosen to avoid the program becoming overly onerous for the schools and teachers implementing the program.

Previous work has shown that the 21-day BDN program is effective in improving children’s toothbrushing knowledge and habits [6]. A study conducted in 10 countries with 7991 children aged between 2 and 12 years revealed that 25% more of the school children brushed their teeth twice a day at the close of the 21-day intervention. The program was found to be more effective among the 7 to 9 year age group.

### Rationale for the Study

The importance of preventing oral diseases to achieve good oral and general health is well known [7]. Regular twice-daily toothbrushing with a fluoride toothpaste is widely recommended for all age groups [8,9] and is effective in improving gingival health and preventing caries. It has been demonstrated that the successful adoption of good brushing habits in childhood can be effective in reducing dental caries risk for the longer term [10].

The 21-day BDN school program is a behavioral change intervention targeting primary school age children [6]. It is an immersive program designed to be delivered by dentists, dental nurses, or teachers at schools. This new study using the 21-day BDN school program aims to build on the previous results [6], with a new design. The previous study used nonprobabilistic convenience sampling with an intervention group only. Additionally, due to differences in evaluation time points which varied between 6 to 12 months, no quantitative conclusions could be drawn from the plaque index score. The study power and robustness of the methodology were cited as necessary improvements in a future investigation.

### Study Objectives

The main objective of the study is to evaluate the impact of a 21-day school program on children’s oral health. The primary objective is to evaluate the impact on knowledge, behavior, and toothbrushing habits in school children after a 21-day school program and compare with baseline and control group. Subsequent objectives will examine the impact on oral health, durability of the intervention, and any wider benefits on parent/carer and child well-being. The specifics of the objectives are outlined in Textbox 1.

**Study objectives.**

- Primary: measure the impact on knowledge, behavior, and toothbrushing habits in school children after a 21-day school program and compare with baseline and control group
- Secondary: measure the impact on oral health via plaque levels at baseline and after a 21-day school program with children and compare with children in their control group
- Tertiary: evaluate the longer term impact of the 21-day program on knowledge, behavior, and oral health in children after a period of 8 weeks and 24 weeks (ie, approximately 7 months in total)
- Quaternary: provide evidence that the 21-day school program is effective in getting parents and carers to also improve their brushing habits and to brush day and night
- Quinary: measure the change in quality of life, well-being, and social measures of school children after a 21-day oral health program

## Methods

### Intervention

Children participating in the 21-day BDN school program are each provided with toothpaste and a toothbrush and follow brushing instruction, supervised brushing, and the singing of songs to facilitate learning the importance of brushing day and night, with stickers and calendars to track progress. A celebration is held at the end of the program with certificates and rewards. The program is supported by colorful materials with bespoke cartoon characters. Parents are provided with educational leaflets.

### Study Design

This study is a 2-arm, superiority cluster randomized trial. Clusters in this study are infant and junior schools in Indonesia and Nigeria. Schools will be matched into pairs by location and then randomized to intervention and control groups using a randomization table.

**Figure 1:**
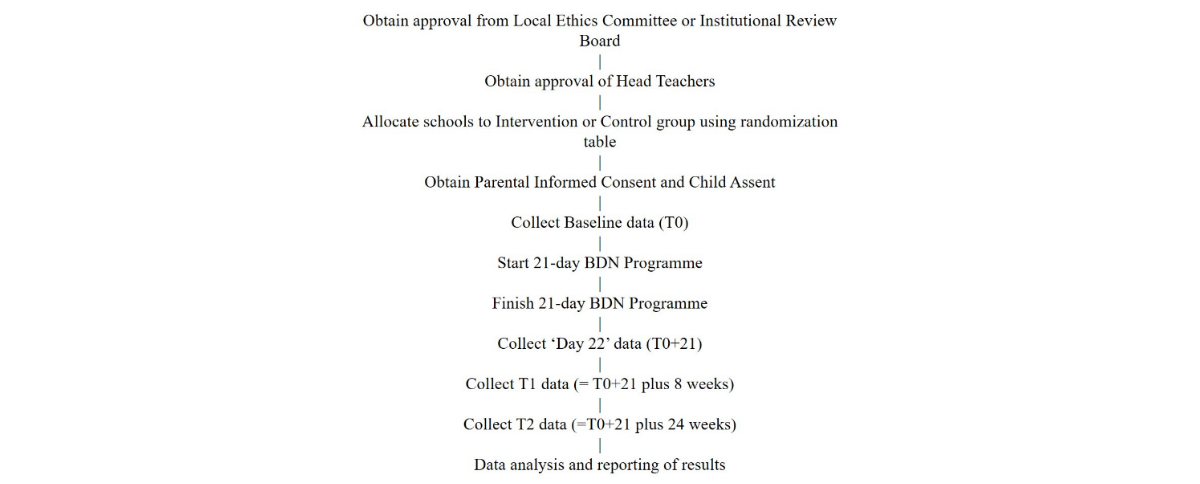
Study flowchart.

The study will look to recruit children aged 6 to 9 years in school grades 1, 2, and 3 to participate in the study. Children who assent to take part in the study (see Multimedia Appendix 2 for form), meet the selection criteria, and have the informed consent of their parents (see Multimedia Appendix 3 for form) will be enrolled in the study. The study flowchart is shown in Figure 1.

The T0 (baseline), T0+21 (end of 21-day intervention), T1 (8 weeks after the end of the intervention), and T2 (24 weeks after the end of the intervention) data collection time points will consist of questionnaire completion and a clinical assessment of plaque level evaluation using the Simplified Oral Hygiene Index (OHI-S) and an additional caries evaluation at T0 and T2 using the Decayed, Missing, and Filled Teeth index (DMFT). For the control group, the same flowchart will be followed, but the “Start 21-day BDN program/Finish 21-day BDN program” is replaced by only providing toothpaste and toothbrushes to school children.

The delivery of the intervention program relies on Unilever materials including toothpaste and toothbrushes as well as all the necessary educational materials. These materials are produced and delivered by Unilever locally. Translations of the materials to a local language will be completed if necessary. Toothpaste provided in Indonesia will be a commercially available and marketed product manufactured by Unilever Indonesia containing 1450 ppm fluoride as 1.12% sodium monofluorophosphate in a chalk base (Pepsodent Anti-Cavity or similar). In Nigeria, a commercially available and marketed toothpaste sold by Unilever Nigeria containing 1450 ppm fluoride (0.32% sodium fluoride) in a silica abrasive (Pepsodent Cavity Fighter Gel or similar) will be provided.

### Selection of Schools

This is a cluster randomization trial with schools being the clusters, randomized to intervention or control groups. Schools need to be matched in terms of setting (city, suburb, rural) and socioeconomic status. The sample size calculation determined the number of schools that would be required to detect a difference between the test and control groups. The response of interest is an improvement in brushing behavior.

For Nigeria, it is assumed that 30% of the children are already brushing twice a day [6]. The trial is designed to detect a minimum 35% difference in the number of children brushing day and night at day 22 between the intervention group and the control group. It is assumed that the control group will see a 20% increase in proportion of children brushing twice a day due to the free toothpaste and toothbrushes they are given (Hawthorne effect). The interclass correlation has been set low at 0.05 and 0.10 (ie, low interpupil correlation) as we do not expect the children to influence each other in their brushing behavior because brushing will be carried out at home.

For Indonesia, it is assumed that 84% of the children are already brushing twice a day [6]. The trial is designed to detect a minimum 30% difference in the number of children brushing day and night at day 22 between the intervention and control group. It is assumed that the control group will have 40% of children brushing twice per day as a result of the Hawthorne effect. Again, the interclass correlation has been set at 0.05.

Therefore, for both Nigeria and Indonesia, the target number of schools is 20 (10 intervention and 10 control schools) calculated using power 80% and a significance level of .05. This number of clusters should also be sufficient to detect a difference of 0.5 in the OHI-S (plaque) at the same power and significance level. About 30 children in each class, in each grade, are anticipated, and for each child, the participation of a parent completing the questionnaires at each time point will be requested.

### Recruitment and Management of Participants

#### Rollout

After written approval from the ethics or education authority has been received, local study coordinators (LSC) will visit each school to obtain the head teacher’s permission to attend schools to deliver the 21-day BDN program and collect data. The LSCs are also responsible for the full delivery of the program from recruiting and matching the schools and composing the dental teams to training the teachers. A dental team is generally composed of 2 to 4 trained and certified dentists and nurses. Dentists will also receive appropriate training and be calibrated for the oral health examinations. A trained and calibrated dental team will be responsible to collect the answers from the self-reported questionnaire and complete the clinical observation of oral health statuses recording the plaque index at all study end points and the caries DMFT index at baseline and at the end of the study (T2). In addition, the LSC will be in charge of training the teachers to deliver the 21-day BDN program over 21 days.

The LSC will be given a list of unique user IDs generated for each school, for each class, and for each child participating in the program. The head teacher will be asked by the LSC to provide a list of the children attending years 1, 2, and 3 in their schools. The list should include first and last name, gender, and date of birth (dd/mm/yyyy) for each child plus the name of the child’s parent.

A member of the study staff will explain the details of the study in person to parents. If parents wish their child to participate, the parent will sign the appropriate informed consent form. Two copies of the informed consent will be completed, one copy will be kept by the parents and one copy returned to the national dental association team. Each LSC is responsible for leading the dental teams. The LSC will assign an ID to each child participating in the study. This ID only plus the exact date of birth will be used to report data, so the data collected will be anonymous once reported.

#### Selection Criteria

A list of inclusion and exclusion criteria will be defined to manage participants in the study. Children in school year grades 1, 2, and 3, aged 6 to 9 years, in good general health, willing and able (eg, to brush teeth and understand and respond to questions) to participate in the 21-day BDN activities at school and at home, and planning on attending their currently registered school for at least the next 12 months will be included.

Exclusion criteria include failure of parents to provide written informed consent, children scheduled for medical or dental procedures during the duration of the study, children who have a known allergy to any toothpaste ingredients, children with obvious signs of gross or untreated caries or of significant periodontal disease which in the opinion of the dentist would affect the scientific validity of the study, or children whose well-being would be affected by the study. Children and their families should have no affiliation (eg, employee) with either FDI or Unilever.

#### Participant Restrictions

A list of restrictions is also set on participants, such as children should not take part in any other form of oral health education for the duration of the study (ie, those that might be delivered at community health centers). Parents should inform their child’s class teacher if they receive any form of dental treatment during the study duration or if they have been prescribed any medicines.

#### Participant Withdrawal

Participants can withdraw from the study at any time, and parents will be advised at the start of the study that they may withdraw their child and family from the study at any time without giving a reason. If a parent withdraws a child from the study, the parent will also be considered withdrawn. Children will be withdrawn from the study analysis if they miss more than 5 days of school during the 21-day intervention period. School attendance during the 21-day period will be checked against the school register.

### Ethics and Quality Standards

The study was presented to the local ethics committees in Nigeria and Indonesia to receive ethics agreements for health research using humans as research subjects. In Nigeria, the study was granted approval by the State Universal Basic Education Board before enrollment of the schools. In Indonesia, the study was granted approval by the Health Research Ethics Commission at the Faculty of Dental Teaching, Trisakti University, also before the enrollment of the schools. The study was registered with ClinicalTrials.gov (NCT04001296).

It is the responsibility of the LSC to ensure that the study is conducted in accordance with the principles of Good Clinical Practice, 2008 version of the Declaration of Helsinki, and applicable local laws and regulations concerning studies conducted on human subjects that are outside of the definition of a medicinal product or medical device. Quality assurance audits may be performed by the sponsor or any ethics committee or regulatory authority during the course of the study or at study completion.

### Study End Points

#### Assessment of Behavioral Change Impact

The objective of this study is to evaluate the impact of educational school programs in improving the oral health of local communities. The primary objective of the study analysis will be to measure the impact on knowledge, behavior, and toothbrushing habits in school children in Indonesia and Nigeria after a 21-day school program. This will be evaluated using three questions (specifically questions on behavior and knowledge) from the children’s questionnaire at all evaluation points (T0, T0+21, T1, T2). Improvement in knowledge and behavior will be calculated based on the percentage of positive change. Positive change is considered when a child selects the correct answers of twice daily brushing with a fluoride toothpaste.

The secondary objective employs the use of the OHI-S to assess plaque levels. More frequent brushing will result in lower plaque levels compared with baseline and compared with control groups. Plaque levels will be used to validate reported brushing frequency with assessments timed to align with questionnaire completion (how well are the children retaining and acting on the information/motivation delivered by the 21-day intervention?). To evaluate the longer term impact of the 21-day program on knowledge/behavior and oral health in children after periods of 8 weeks and 24 weeks, the children in the test group should report higher levels of brushing frequency and reduced plaque levels compared with children in the control group. To provide evidence that the 21-day school program is effective in getting parents and carers to also improve their brushing habits and to brush day and night, parents’ own brushing frequency, use of fluoride toothpaste, amount of toothpaste, and toothbrushes purchased should increase when compared with baseline and compared with parents in the control group. To measure the change in quality of life, well-being, and social measures of school children after a 21-day oral health program, children may report less dental discomfort/pain and need to take fewer days off school as a result. Additionally, at baseline (T0) and T2, all children will be scored for their caries prevalence using the DMFT index. No direct conclusions on the caries can be made but it is important descriptive information that is often not available in these two countries and could serve further research.

#### Questionnaires

This study aims to understand, to a greater extent, the impact of the 21-day BDN program on quality of life–related outcomes and, thus, includes more detailed questions and addresses some aspects of the new oral health definition [11].

The parent questionnaire includes 14 main questions addressing aspects on oral health behavior and knowledge, socioeconomic factors, well-being, and quality of life–related outcomes. Most of the parent questions were taken from validated questionnaires, namely the World Health Organization oral health surveys, Global Oral Health Assessment Index, and Positive Oral Health and Well-being 15-term [12-14]. Other questions were developed through a process in which an expert group identified topics included in similar surveys and developed questions on these topic areas adapted to the study objectives and setting. Parent questions can be found in Multimedia Appendix 4.

The children’s questions were derived from the parent questions and adapted for children’s comprehension and to obtain the required information as set by the objectives of the study. In addition, building on learning gained in the previous study [6], the questionnaire was further refined to remove overlapping answers encountered that required some retrospective reinterpretation prior to analysis.

The children’s questionnaire includes 8 main questions to address the primary objective of improvement in behavior and knowledge, adapted to their comprehension.

- Did you brush your teeth yesterday?
- If yes, how many times?
- Did you brush your teeth today?
- If yes, how many times?
- How often do you brush your teeth at home?
- Most days, do you brush your teeth both in the morning and the evening?
- Do you think it is important to brush your teeth every day?
- If yes, how often should you brush your teeth every day?

An intense immersion into a fun and engaging 21-day BDN program will inform children of the importance of twice a day brushing with a fluoridated toothpaste and motivate improved brushing frequency. This will be seen in results of questionnaires issued to children at time points throughout the study. All children’s questions can be found in Multimedia Appendix 5.

#### Simplified Oral Hygiene Index

The OHI-S [15] is a rapid method for evaluating oral cleanliness of population groups. In this study, the following permanent teeth will be scored: 16, 11, 26, 46, 31, and 36. However, due to the specific target age group of the study, the equivalent deciduous teeth 55, 51, 65, 85, 71, and 75 will be scored when the above-mentioned permanent teeth are not present.

#### Decayed, Missing, and Filled Teeth Index: Tooth Level

The DMFT index [14] has been used for more than 70 years and is well established as the key measure of caries experience in dental epidemiology. The DMFT index can be applied to both the permanent and primary dentition. Table 1 shows the full schedule of study end points by time point and intervention for this study. The schedule is the same for control and intervention groups.

**Table 1:** Study end points by time point and intervention.

Group and end point		Baseline T0	T0+21^a^	T1^b^	T2^c^
**Children**					
	Questionnaire	x	x	x	x
	OHI-S^d^	x	x	x	x
	DMFT^e^	x			x
**Parents/carers**					
	Questionnaire	x	x	x	x

^a^T0+21: end of 21-day intervention.

^b^T1: 8 weeks after the end of the intervention.

^c^T2: 24 weeks after the end of the intervention.

^d^OHI-S: Simplified Oral Hygiene Index.

^e^DMFT: Decayed, Missing, and Filled Teeth index.

### Data Management

#### Data Exclusion

All school data will be used, and where a school has been significantly noncompliant (eg, a school has not sufficiently followed and completed all elements of the 21-day BDN program) with the protocol, this will be registered. Minor deviations may be included at the discretion of the LSC. Participants who missed more than 5 days (one school week) of the 21-day BDN program will have this information registered in the analysis.

#### Data Handling and Record Keeping

Paper forms (Multimedia Appendix 6) will be used to collect all questionnaire answers from parents and children as well for reporting the oral health indicators used, the OHI-S and DMFT. All collected data will be compiled using SPSS Statistics (IBM Corp) software for study analysis.

The LSC will keep a separate confidential enrollment log that matches identifying codes with children’s names and addresses. The LSC will maintain these documents at the site. It is the responsibility of the LSC or designee to maintain adequate clinical study records. Copies of all clinical study material must be archived for a period of at least 15 years after the end of the study (or more as legally required). All documents must be archived in a secure place and treated as confidential material. The anonymous data will be made available to researchers upon request.

### Statistical Analysis

As outlined in Table 2, five outcomes measures have been prepared and prioritized to evaluate the effect of the 21-day program. At each time point, summary statistics will be calculated for intervention and control schools and include within- and between-group analyses.

Each of the mentioned measures in Table 2 will be compared between T0 and T0+21 and between T0 and T0+21 and T1 and T2 for the intervention and control groups. The mean change will be reported, with 95% confidence interval and associated *P* value. Longitudinal models will be used. Multinomial logistic regression will be performed when the outcome variables have a nominal level of measurement. For ordinal and interval outcome variables, we will use either ordered logistic or linear regression as appropriate. To measure positive change, we will convert nominal or ordinal independent variables using dummy variables where each dummy will be measuring the effect of one answer category on the reference category.

Each of the above measures will be compared between the intervention and control group at T0, T0+21, T1, and T2 using analyses of covariance, mixed models, and generalized estimating equations as appropriate. Mean differences will be reported, with 95% confidence interval and associated *P* value.

In addition to analyzing the impact of the program on various beneficiaries through behavior and health change, the global impact of the 21-day program will be analyzed. This analysis will be performed by multivariate analysis, which measures the influence of each outcome variable on the others and of each independent variable on the relevant outcome variables. We will also measure some of the variables with a measurement model such as factor analysis. All statistical analyses will be performed using SPSS Statistics software.

To minimize bias in the results, the intention to treat principle will be applied and every randomized participant will be analyzed. The school structure is helping us to minimize missing data, but it may still occur due to the longitudinal aspect of the study and number of follow-ups. In this case, carrying the last data forward should be the best approach.

**Table 2:** Study objectives as they relate to the statistical analysis plan.

Objective	Outcome variables	Results observed for	Hypotheses
Primary: measure the impact on knowledge/behavior and toothbrushing habits in school children after a 21-day school program and compare with baseline and a control group	Child questionnaire: reported brushing frequency, knowledge of the need to brush twice a day, reported use of fluoride toothpaste	within-group analysis: T0+21 vs T0; between-group analysis: T0, T0+21	Immersion in a structured 21-day school program will inform children of the importance of twice a day brushing with a fluoridated toothpaste and motivate improved brushing frequency.
Secondary: measure the impact on oral health via plaque levels in school children after a 21-day school program and compare with baseline and a control group	Child clinical observation: plaque levels (OHI-S^a^) will be used to validate reported brushing frequency with assessments timed to align with questionnaire completion (how well are the children retaining and acting on the information/motivation delivered by the 21-day intervention?)	within-group analysis: T0+21 vs T0; between-group analysis: T0, T0+21	More frequent brushing will result in lower plaque levels compared with baseline and compared with their control group.
Tertiary: evaluate the longer term impact of the 21-day program on knowledge/behavior and oral health in children after a period of 8 weeks (T1) and 24 weeks (T2) or approximately 7 months total	Child questionnaire: reported brushing frequency at T1 and T2; knowledge of the need to brush twice a day and reported use of fluoride toothpaste at T1 and T2; and OHI-S at T1 and T2 compared with baseline, after 21 days, and with control group	within-group analysis: T2 vs T0, T0+21, and T1 vs T0, T0+21; between-group analysis: T0, T0+21, T1, T2	Children in the intervention group will report higher levels of brushing frequency and reduced plaque levels compared with the children in the control group. This can be attributed to the 21-day intervention.
Quaternary: provide evidence that the 21-day school program is effective in getting parents and carers to also improve their brushing habits and to brush day and night.	Parent questionnaire: reported brushing frequency, reported use of fluoride, renewing toothbrush frequency, amount of purchased toothpaste	within-group analysis: T0+21 vs T0; between-group analysis: T0, T0+21	Consenting for their children to participate in a toothbrushing program will likely trigger a reflection upon their own oral care habits, and parents’ own brushing frequency, use of fluoride toothpaste, amount of toothpaste, and toothbrushes purchased should increase when compared with baseline and compared with parents in the control group.
Quinary: measure the change in quality of life, well-being, and social measures of school children after a 21-day oral health program	Child questionnaire: reported absenteeism for oral health issues	within-group analysis: T0+21 vs T0; between-group analysis: T0, T0+21	Children may report less dental discomfort/pain and a need to take fewer days off school as a result.

^a^OHI-S: Simplified Oral Hygiene Index.

### Safety Monitoring

#### Adverse Event

An adverse event (AE) will be considered as any untoward medical occurrence in a subject that is new in onset or an exacerbation of a preexisting condition, whether related to study product or procedures or not. However, medical occurrence resulting from a pretreatment AE (ie, any medical occurrence that occurs after informed consent but before the start of the intervention) will be considered as medical history and only recorded as an AE if it worsens during the study. Similarly, a medical occurrence resulting from preexisting medical condition (ie, events that occur with comparable frequency and severity to the subject’s baseline condition) is reported as medical history and not AE. Relatedness—the likelihood that an AE is related to the study product or study procedure—of an AE is defined in Table 3. An AE will be recorded only once, with the most extreme severity:

- Mild: awareness of symptoms that require minimal or no treatment and do not interfere with daily activity
- Moderate: discomfort or low level of interference that is enough to interfere with but not prevent daily activity
- Severe: interrupted or unable to perform usual daily activity; usually requires treatment

**Table 3:** Relatedness of adverse events.

Relatedness	Definition
Not related	AE^a^ is clearly due to an alternative cause, even if this cannot be definitely identified. Alternative causes include diseases and environmental factors.
Unlikely	Connection between AE and the study product or procedure is unlikely: AE has a relationship in time to the study product or procedure Alternative cause (eg, disease or environmental factor) is the most likely explanation, even if this cannot be identified
Possibly	Connection between AE and the study product or procedure cannot be ruled out with certainty: AE has a relationship in time to the study product or procedure Alternative cause (eg, disease or environmental factor) seems likely or possible or there is significant uncertainty about the cause of the AE
Probably	There is a high degree of certainty that the AE is related to the study product or procedure: AE has a relationship in time to the study product or procedure Possible alternative cause may be present AE disappears or decreases on withdrawal or reduction of study product or procedure (if performed)
Definitely	AE is clearly related to the study product or procedure: There is a strong relationship in time Alternative cause is unlikely AE disappears or decreases on withdrawal or reduction of study product or procedure (if performed)

^a^AE: adverse event.

#### Serious Adverse Event

A serious adverse event (SAE) is an AE that results in any of the following outcomes: death, life-threatening event, in-patient hospitalization, persistent or significant disability/incapacity. Any other important medical event may be considered an SAE when the event may jeopardize the subject and may require medical or surgical intervention to prevent one of the outcomes listed. A stable preexisting condition is not an AE, and hospitalization for elective treatment (eg, cosmetic or dental procedure) of a preexisting condition that did not worsen from baseline is not an SAE.

#### Reporting of Adverse Events

AEs will be monitored, reported, and followed up to the public health authorities. All AEs will be recorded and submitted to the FDI global team at the end of the study. The LSC will maintain source documents to fully record all AEs.

Additionally, SAEs and clusters of AEs that may affect the safety or continued participation of participants in the study will be reported immediately using a more detailed form. An SAE will be reported to FDI within 24 hours of the LSC becoming aware of the event.

#### Follow-Up of Adverse Events

If an AE is ongoing at the end of the study, follow-up will be performed until the AE has resolved, unless decided and agreed that no further follow-up will be necessary. Follow-up may take the form of subject visits, referral to another specialist, site telephone calls to the subject, or letters from the treating physician. For expedited AEs, if applicable, the LSC will submit follow-up reports. The LSC will comply with the specific reporting requirements of the ethics committee, reporting as a minimum any serious unexpected adverse reaction that is an unexpected SAE that may be related to study product or procedure.

## Results

This protocol is being retrospectively published with the information shared with the two participating countries on January 19, 2019. In the meantime, the trial was set up, and the study is ongoing. Recruitment of schools and participants started in Indonesia in February 2018 and in Nigeria in April 2018 for the first part of the study, which concluded in Indonesia in September 2018 and in Nigeria in November 2018. The second part of the study (the second half of the schools) started in November 2018 in Indonesia and December 2018 in Nigeria. We expect to collect all the data during 2019 and publish findings from the study by March 2020.

## Discussion

This new study will include both intervention and control schools and children and ensure that evaluations (knowledge, behavior, plaque levels, and dental caries) are made within a clearly defined time window to facilitate data analysis.

Children in the control group will not receive the 21-day school program intervention. This will allow a full evaluation of the benefit of the intervention. The OHI-S will be used to score plaque. This is a 4-point scale and hence will be more discriminatory than the 2-point (visible plaque index) scale used previously. This is the first time that the 21-day BDN program has been evaluated in a study with a set of control schools. The impact of such a program on parents is also being studied for the first time. We expect this study will increase knowledge of the effectiveness of this intervention in bringing immediate and sustained change in knowledge, behavior, toothbrushing habits, oral health, quality of life, and well-being in children aged 6 to 9 years and their parents. We aim to publish the results in a special edition of the International Dental Journal by March 2020. In addition, results will be presented at conferences and disseminated among project leaders as guidance for the development and continuation of implementation of school programs in their countries.

## Appendix

Multimedia Appendix 1

Multimedia Appendix 2 Child assent form—English.

Multimedia Appendix 3 Parent informed consent form—English.

Multimedia Appendix 4 Parent questionnaire—English.

Multimedia Appendix 5 Child questionnaire—English.

Multimedia Appendix 6 Decayed missing filled tooth index and simplified oral hygiene index: paper form for data collection.

## Abbreviations

AE: adverse event
BDN: Brush Day & Night
DMFT: Decayed, Missing, and Filled Teeth Index
FDI: FDI World Dental Federation
LSC: local study coordinator
OHI-S: Simplified Oral Hygiene Index
SAE: serious adverse event
T0: baseline
T0+21: end of 21-day intervention
T1: 8 weeks after the end of the intervention
T2: 24 weeks after the end of the intervention
