# Supplementary material for: A 21-Day School-Based Toothbrushing Intervention in Children Aged 6 to 9 Years in Indonesia and Nigeria: Protocol for a Two-Arm Superiority Randomized Controlled Trial
Source: JMIR Res Protoc. 2020 Feb 21;9(2):e14156. doi: 10.2196/14156 (PMC7060496; doi:10.2196/14156)
Supplement: Multimedia Appendix 1 [file resprot_v9i2e14156_app1.docx]

**Multimedia Appendix 1. 21-day Brush Day & Night programme**

The 21-day Brush Day & Night Programme is an intense education and activity programme to establish the habit of brushing day and night which has been developed on the principles of Unilever’s Behaviour Change model[13]. The programme is implemented with the support of a team, including 2 to 4 dentists and 1 or 2 nurses and teachers to the target age range children of 6-9 years old. It aims to empower children to become advocates of twice daily brushing with fluoride toothpaste with their parents, families and communities. Aligning with the 5 levers of Behaviour Change Model, there are 5 steps to the programme as detailed below:

1. Make it understood. Dentists/teachers explain oral hygiene with flipcharts and demonstrate the proper way to brush your teeth.
2. Make it desirable. The Brush Day and Night song and pledge are introduced. The song and pledge are repeated daily. Supporting materials include cartoon characters that are attractive to children.
3. Make it easy. The children are provided with toothbrush and toothpaste samples to encourage practice at home. An educational leaflet for parents and carers is sent home.
4. Make it a habit. A sticker calendar for children and adults allows twice daily brushing to be recorded. The parents sign every day that the brushing habit has been done by both adults and children.
5. Make it rewarding. Daily rewards and a ‘graduation’ celebration complete the programme.

Participating schools assigned to the Intervention group will be provided with the following materials and resources:

- Trained Dentists/Nurses to deliver the programme
- Toothpaste and toothbrushes
  - In Indonesia: A commercially available and marketed toothpaste manufactured by Unilever Indonesia containing 1450ppm Fluoride as 1.12% sodium monofluorophosphate in a chalk base, e.g. Pepsodent Anti-Cavity or similar
  - In Nigeria: A commercially available and marketed toothpaste sold by Unilever Nigeria containing 1450ppm Fluoride (0.32% Sodium Fluoride) in a silica abrasive, e.g. Pepsodent Cavity Fighter Gel or similar
- Brush Day & Night cartoon themed and branded. Leaflets, calendars, stickers, certificates.

Schools/children in the Control group will just receive toothpaste and toothbrushes and no Intervention.
