# Supplementary material for: A 21-Day School-Based Toothbrushing Intervention in Children Aged 6 to 9 Years in Indonesia and Nigeria: Protocol for a Two-Arm Superiority Randomized Controlled Trial
Source: JMIR Res Protoc. 2020 Feb 21;9(2):e14156. doi: 10.2196/14156 (PMC7060496; doi:10.2196/14156)
Supplement: Multimedia Appendix 2 [file resprot_v9i2e14156_app2.docx]

**Multimedia Appendix 2. Children assent form – English**

**Child Assent Form for the FDI / Unilever phase IV protocol.**

**STUDY NUMBER: *FDIUL-BDN-2018-01***

**Study title:**

**A study to examine the impact of a 21-day school brushing intervention on the knowledge, behaviour and oral health of school children**

We’re doing a study to find out how often you brush your teeth and how to motivate you to keep a healthy mouth.

If you would like to participate in this study, you need to understand what you’ll have to do. We’ll ask you to answer to some questions about your teeth and how often you brush them (your parent or teacher will help you with this). A dentist will look inside your mouth to see if your teeth are strong and healthy. This may take a few minutes and you’ll need to be still and keep your mouth open during this time.

For three weeks your teacher will show you how to brush and how often you should brush your teeth and give you tips to keep your teeth strong and healthy.

We’ll give you free toothpaste and toothbrushes for 2018 and 2019 during your participation in this study.

We’ll be asking all your classmates if they want to participate as well. If at any time you don’t want to continue, just let your teacher know. We’ll also ask permission from your parent/carer/guardian for you to participate.

Do you understand what this project is about? Yes/No

If you had any question, did you understand the answers? Yes/No

Are you happy to participate? Yes/No

If ANY of your answers are ‘No’ or you don’t want to take part, then don’t write your name!

If you DO want to take part, you can write your name below:

Your name….……………………………….… Date (DD/MM/YYYY) ……………………………..

For Study Staff use only:

Consent Administered by: (print name) ………………………………………………………………………………………………..………….

Signature……………………………… Date (DD/MM/YYYY) ………………………..
