# Supplementary material for: A 21-Day School-Based Toothbrushing Intervention in Children Aged 6 to 9 Years in Indonesia and Nigeria: Protocol for a Two-Arm Superiority Randomized Controlled Trial
Source: JMIR Res Protoc. 2020 Feb 21;9(2):e14156. doi: 10.2196/14156 (PMC7060496; doi:10.2196/14156)
Supplement: Multimedia Appendix 3 [file resprot_v9i2e14156_app3.docx]

**Multimedia Appendix 3. Parents informed consent form – English**

**Parental / Carer / Guardian Informed Consent Forms for the FDI / Unilever phase IV protocol.**

**STUDY NUMBER: *FDIUL-BDN-2018-01***

**Study title:**

**A study to examine the impact of a 21-day school brushing intervention on the knowledge, behaviour and oral health of school children**

**Introduction** You and your child are being asked to take part in a research study. Before you agree, please take the time to carefully understand what your participation will involve.

Once you’ve read and understood this information, you can decide whether or not to take part in the study. If you agree to participate, you will receive a signed and dated copy of this information sheet/consent form (all pages) before the study begins. You will also be given any other written information you might need to participate.

**Why are we doing this study?** We’re doing this study to see how effective a 21-day ‘Brush Day and Night’ programme run in schools is at improving brushing behaviour and knowledge among children.

**What’s involved?** You’re being asked to give permission for your son/daughter and yourself to take part in a 28 weeks study. All the other parents/guardians with children in the same class at school are also being asked to take part in the study. During school time, your child will receive instructions and daily reminders on how to brush their teeth and the importance of brushing twice a day with a fluoride toothpaste. Their teachers will lead the activity, providing the children with a tube of toothpaste, a toothbrush, calendars and stickers to help them remember to brush twice a day.

We’ll also ask you about how you feel about your oral health and your oral care habits.

If you and your child would like to join the study, you will each be asked to complete a short questionnaire on four different occasions. Additionally, the children will have their mouths examined by a dentist on four occasions. During the examinations, the dentist will be looking for cavities and plaque build-up on the teeth

| Time point | Child | Parent |
| --- | --- | --- |
| 1, T0 in February 2018 | Questionnaire, mouth examination (cavities and plaque) | Questionnaire |
| 2, T0+21 in March 2018 | Questionnaire, mouth examination (plaque only) | Questionnaire |
| 3, T1 in May 2018 | Questionnaire, mouth examination (plaque only) | Questionnaire |
| 4, T2 in August/September 2018 | Questionnaire, mouth examination (cavities and plaque) | Questionnaire |

**What are the benefits?** Your child will receive free tubes of toothpaste with fluoride and toothbrushes. In addition, your child will receive a calendar and stickers to help them remember to brush twice a day.

We hope that by taking part in this study your child will develop the habit of brushing twice a day. Twice a day brushing is recommended by dentists as key to good oral health throughout life

**What are the risks?** The toothpaste provided is already available in the shops in Indonesia/Nigeria. Hence, we do not anticipate any problems in using the toothpaste. If you or your family do have a problem, you can contact:

| Name: | Role: | Phone: |
| --- | --- | --- |

The mouth examinations will be carried out by qualified and experienced dentists. The child will need to be still and open their mouths wide to allow the examination to take place. The examination should not cause the child any discomfort.

If you or your child wishes to stop taking part in the study at any time, you may do so at any time. Participation is strictly voluntary.

**Informed Consent**

1. I have fully understood the above information and understand the purpose of the study
2. I have understood the activities that my child and I will be required to participate in and the duration of the study.
3. I have been made aware of the foreseeable risks and benefits of taking part in the study.
4. I know I can stop taking part in the study at any time.
5. I have had the opportunity to ask questions, and my questions have been satisfactorily answered.
6. On behalf of my child:

- I confirm that my child is in good general health
- Is currently planning on attending their current school for the next 8 months
- Is not scheduled to undergo any medical or dental procedures during the duration of the study
- Has not previously shown any allergic reaction to any toothpaste ingredients

1. My child and I are willing to take part in the study

| Name of the child |  |
| --- | --- |
| School |  |
| Class |  |
| Name of the Parent / Guardian |  |

Signature of the Parent / Guardian………………………Date (DD/MM/YY).…………………….

For Study Staff use only:

Consent Administerd by: (print name) ……………………………………………………………………………………………………………

Signature…………………………………………Date (DD/MM/YYYY) ……………………………

**Photographic Consent Statement**

I give permission for my child to be photographed, either still or moving, while participating in the study. I understand that my child’s personal information will never be published with his/her image. I authorize FDI and Unilever to use my child’s image for the following:

- Printed and digital materials promoting the project, Brush Day & Night (including social media)
- Printed and digital materials in support of the aims and objectives of the organization/company
- Printed and digital materials for internal communications, such as reports and presentations

Signature…………………………………………Date (DD/MM/YYYY) ……………………………
