# Supplementary material for: A 21-Day School-Based Toothbrushing Intervention in Children Aged 6 to 9 Years in Indonesia and Nigeria: Protocol for a Two-Arm Superiority Randomized Controlled Trial
Source: JMIR Res Protoc. 2020 Feb 21;9(2):e14156. doi: 10.2196/14156 (PMC7060496; doi:10.2196/14156)
Supplement: Multimedia Appendix 4 [file resprot_v9i2e14156_app4.docx]

**Multimedia Appendix 4. Questionnaire addressed to parents - English**

| **What to measure and related indicators** | **PARENTS QUESTIONS** | | | | | | |
| --- | --- | --- | --- | --- | --- | --- | --- |
|  |  |  |  |  |  |  |  |
|  |  |  |  |  |  |  |  |
| **Age** | **Q1** | **How old are you?** | | | | | |
|  |  |  |  | [Input Field] | | | |
|  |  |  |  |  |  |  |  |
| **Gender** | **Q2** | **What is your gender?** | | | | | |
|  |  |  | **1** | Male | | | |
|  |  |  | **2** | Female | | | |
|  |  |  | **99** | I'd prefer not to say | | | |
|  |  |  |  |  |  |  |  |
| **A’= Oral health related to quality of life/well-being** -self-rated oral Health | **Q3** | **How would you rate your oral health?** | | | | | |
|  |  |  | **1** | Very poor | | | |
|  |  |  | **2** | Poor | | | |
|  |  |  | **3** | Fair | | | |
|  |  |  | **4** | Good | | | |
|  |  |  | **5** | Very good | | | |
|  |  |  |  |  |  |  |  |
| **A’= Oral health related to quality of life/well-being** -eating | **Q4** | **Have you had difficulty eating food, including difficulties chewing or biting, due to problems with your mouth, teeth or dentures during the past 12 months?** | | | | | |
|  |  |  | **1** | Yes |  |  |  |
|  |  |  | **2** | No |  |  |  |
|  |  | **Q4.1** | **[If yes] - How much has it affected your daily life on a scale of 1-5, with 1 being not at all and 5 being very much?** | | | | |
|  |  |  |  | [Input Field] | | | |
|  |  |  |  |  |  |  |  |
| **A’ = Oral health related to quality of life/well-being** -smiling | **Q5** | **Have you been embarrassed to smile or laugh because of your mouth, teeth or dentures during the past 12 months?** | | | | | |
|  |  |  | **1** | Yes |  |  |  |
|  |  |  | **2** | No |  |  |  |
|  |  | **Q5.1** | **[If yes] - How much has it affected your daily life on a scale of 1-5, with 1 being not at all and 5 being very much?** | | | | |
|  |  |  |  | [Input Field] | | | |
|  |  |  |  |  |  |  |  |
| **A’ = Oral health related to quality of life/well-being** -General Well-being | **Q6** | **My oral health has a good impact on my general well-being:** | | | | | |
|  |  |  | **1** | Strongly disagree |  |  |  |
|  |  |  | **2** | Disagree |  |  |  |
|  |  |  | **3** | Agree |  |  |  |
|  |  |  | **4** | Strongly Agree |  |  |  |
|  |  |  | **99** | Neutral/Do not know |  |  |  |
|  |  |  |  |  |  |  |  |
| **B’ = Behaviour change** -Toothbrushing timing and frequency | **Q7** | **How often do you brush your teeth?** | | | | | |
|  |  |  | **1** | 1 time per day |  |  |  |
|  |  |  | **2** | 2 times per day |  |  |  |
|  |  |  | **3** | 3 or more times a day |  |  |  |
|  |  |  | **4** | A few times per week |  |  |  |
|  |  |  | **5** | 1 time per week |  |  |  |
|  |  |  | **6** | 2-3 times a month |  |  |  |
|  |  |  | **7** | Less than 2 times a month |  |  |  |
|  |  |  | **8** | Irregularly |  |  |  |
|  |  |  | **9** | Never/Do not brush | | | |
|  |  | **Q7.1** | **[If answers 1-8] - When do you brush your teeth? (select all that apply):** | | | | |
|  |  |  | **1** | Morning before breakfast |  |  |  |
|  |  |  | **2** | Morning after breakfast |  |  |  |
|  |  |  | **3** | After lunch |  |  |  |
|  |  |  | **4** | Evening but eat after brushing | | | |
|  |  |  | **5** | Evening and no eating after brushing | | | |
|  |  |  | **6** | Other time of day |  |  |  |
|  |  |  | **99** | Do not know |  |  |  |
|  |  | **Q7.2** | **How long do you usually brush your teeth for?** | | | | |
|  |  |  | **1** | Less than 30 seconds |  |  |  |
|  |  |  | **2** | 30 seconds to 1 minute |  |  |  |
|  |  |  | **3** | 1 minute or more but less than 2 minutes |  |  |  |
|  |  |  | **4** | 2 minutes or more but less than 3 minutes | | |  |
|  |  |  | **5** | 3 minutes or more |  |  |  |
|  |  |  | **99** | D o not know |  |  |  |
|  |  | **Q7.3** | **What do you use to brush your teeth?** | | | | |
|  |  |  | **1** | Toothbrush and toothpaste |  |  |  |
|  |  |  | **2** | Toothbrush |  |  |  |
|  |  |  | **3** | Chewing stick/Miswak |  |  |  |
|  |  |  | **4** | Charcoal |  |  |  |
|  |  |  | **5** | Other |  |  |  |
|  |  |  | **99** | Do not know |  |  |  |
|  |  | **Q7.4** | **How frequently do dentists recommend you should brush your teeth?** | | | | |
|  |  |  | **1** | Once a day |  |  |  |
|  |  |  | **2** | Twice a day |  |  |  |
|  |  |  | **99** | I don't know |  |  |  |
|  |  |  |  |  |  |  |  |
| **B’ = Behaviour change** -usage of fluoride | **Q8** | **[if Q7.3 answer A], do you use fluoride toothpaste?** | | | | | |
|  |  |  | **1** | Yes, for me and my children |  |  |  |
|  |  |  | **2** | Yes, only for me |  |  |  |
|  |  |  | **3** | Yes, only for my children |  |  |  |
|  |  |  | **4** | No |  |  |  |
|  |  |  | **99** | Do not know |  |  |  |
|  |  |  |  |  |  |  |  |
| **B’ = Behaviour change** -home care habits | **Q9** | **How often do you change your toothbrush for a new one?** | | | | | |
|  |  |  | **1** | More frequently than once a month |  |  |  |
|  |  |  | **2** | Once a month |  |  |  |
|  |  |  | **3** | Every 2 months |  |  |  |
|  |  |  | **4** | Every 3 months |  |  |  |
|  |  |  | **5** | More than every 3 months |  |  |  |
|  |  |  | **99** | I don't know / can't remember |  |  |  |
|  |  | **Q10.1** | **With how many people do you share the toothbrush?** | | | | |
|  |  |  | **1** | Only me |  |  |  |
|  |  |  | **2** | With 1 person |  |  |  |
|  |  |  | **3** | With 2 people |  |  |  |
|  |  |  | **4** | With 3 people |  |  |  |
|  |  |  | **5** | With 4 people |  |  |  |
|  |  |  | **6** | With 5 people |  |  |  |
|  |  |  | **7** | With 6 or more people |  |  |  |
|  |  |  |  |  |  |  |  |
| **B’ = Behaviour change** -home care habits | **Q10** | **How often do you buy new toothpaste?** | | | | | |
|  |  |  | **0** | More frequently than once a month |  |  |  |
|  |  |  | **1** | Once a month |  |  |  |
|  |  |  | **2** | Every 2 months |  |  |  |
|  |  |  | **3** | Every 3 months |  |  |  |
|  |  |  | **4** | More than every 3 months |  |  |  |
|  |  |  | **99** | I don't know / can't remember |  |  |  |
|  |  | **Q9.1** | **With how many people do you share the toothpaste?** | | | | |
|  |  |  | **0** | Only me |  |  |  |
|  |  |  | **1** | With 1 person |  |  |  |
|  |  |  | **2** | With 2 people |  |  |  |
|  |  |  | **3** | With 3 people |  |  |  |
|  |  |  | **4** | With 4 people |  |  |  |
|  |  |  | **5** | With 5 people |  |  |  |
|  |  |  | **6** | With 5 or more people |  |  |  |
|  |  |  |  |  |  |  |  |
| **B’ = Behaviour change** -home care habits | **Q11** | **Do you use mouthwash?** | | | | | |
|  |  |  | **1** | Yes |  |  |  |
|  |  |  | **2** | No |  |  |  |
|  |  | **Q11.1** | **if yes, how often do you buy new bottles of mouthwash?** | | | | |
|  |  |  | **1** | More frequently than once a month |  |  |  |
|  |  |  | **2** | Once a month |  |  |  |
|  |  |  | **3** | Every 2 months |  |  |  |
|  |  |  | **4** | Every 3 months |  |  |  |
|  |  |  | **5** | More than every 3 months |  |  |  |
|  |  |  | **99** | I don't know / can't remember |  |  |  |
|  |  | **Q11.2** | **With how many people do you share the mouthwash?** | | | | |
|  |  |  | **1** | Only me |  |  |  |
|  |  |  | **2** | With 1 person |  |  |  |
|  |  |  | **3** | With 2 people |  |  |  |
|  |  |  | **4** | With 3 people |  |  |  |
|  |  |  | **5** | With 4 people |  |  |  |
|  |  |  | **6** | With 5 people |  |  |  |
|  |  |  | **7** | With 6 or more people |  |  |  |
|  |  |  |  |  |  |  |  |
| **C’ = Socio-economic factors** -Missed days from work | **Q12** | **If you work, have you taken time off because of problems related to your mouth, teeth or dentures during the past 12 months?** | | | | | |
|  |  |  | **1** | Yes |  |  |  |
|  |  |  | **2** | No |  |  |  |
|  |  |  | **99** | Not applicable |  |  |  |
|  |  | **Q12.1** | **[If yes] - Approximately how many days off have you taken during the last 12 months?** | | | | |
|  |  |  |  | [Input Field] | | | |
|  |  |  |  |  |  |  |  |
| **Social interaction** | **Q13** | **Have you limited participation in social activities, or had any difficulty enjoying the contact of other people, due to problems with your mouth, teeth or dentures during the past 12 months?** | | | | | |
|  |  |  | **1** | Yes |  |  |  |
|  |  |  | **2** | No |  |  |  |
|  |  | **Q13.1** | **[If yes] - How much has it affected your daily life on a scale of 1-5, with 1 being not at all and 5 being very much?** | | | | |
|  |  |  |  | [Input Field] | | | |
|  |  |  |  |  |  |  |  |
| **C’ = Socio-economic factors** -level of education | **Q14** | **What is the highest level of education you have completed?** | | | | | |
|  |  |  | **0** | No formal education |  |  |  |
|  |  |  | **1** | Early childhood education |  |  |  |
|  |  |  | **2** | Primary education |  |  |  |
|  |  |  | **3** | Lower secondary education |  |  |  |
|  |  |  | **4** | Upper secondary education |  |  |  |
|  |  |  | **5** | Post-secondary non-tertiary education |  |  |  |
|  |  |  | **6** | Short-cycle tertiary education |  |  |  |
|  |  |  | **7** | Bachelor’s or equivalent level |  |  |  |
|  |  |  | **8** | Master’s or equivalent level |  |  |  |
|  |  |  | **9** | Doctoral or equivalent level |  |  |  |
