# Supplementary material for: A 21-Day School-Based Toothbrushing Intervention in Children Aged 6 to 9 Years in Indonesia and Nigeria: Protocol for a Two-Arm Superiority Randomized Controlled Trial
Source: JMIR Res Protoc. 2020 Feb 21;9(2):e14156. doi: 10.2196/14156 (PMC7060496; doi:10.2196/14156)
Supplement: Multimedia Appendix 5 [file resprot_v9i2e14156_app5.docx]

## Multimedia Appendix 5. Questionnaire addressed to children – English

| **What to measure and related indicators** | **CHILDREN QUESTIONS** | | | | | |
| --- | --- | --- | --- | --- | --- | --- |
|  |  |  |  |  |  |  |
|  |  |  |  |  |  |  |
| **Age** | **Q1** | **What is your date of birth?** | | | | |
|  |  |  | **1** | DD/MM/YYYY | | |
|  |  |  |  |  |  |  |
| **Gender** | **Q2** | **What is your gender?** | | | | |
|  |  |  | **1** | Male | | |
|  |  |  | **2** | Female | | |
|  |  |  |  |  |  |  |
| **A’= Oral health related to quality of life/well-being** -Eating | **Q3** | **Does it hurt in your mouth when you eat something?** | | | | |
|  |  |  | **1** | Yes | | |
|  |  |  | **2** | No | | |
|  |  | **Q3.1** | ** [If yes] - What do you feel? Rate using the 3 pain scale faces*** | | | |
|  |  |  | **1** | It hurts | **** | |
|  |  |  | **2** | Make me cry |  |  |
|  |  |  | **3** | Hurts a little bit / sometimes |  |  |
|  |  |  | **99** | Not sure/Do not know/ not applicable |  |  |
|  |  |  |  |  |  |  |
| **A’= Oral health related to quality of life/well-being** -smiling/confidence | **Q4** | **Are you happy to give a big smile when playing with your friends?** | | | | |
|  |  |  | **1** | Yes |  |  |
|  |  |  | **2** | No | | |
|  |  |  | **99** | Not sure/Do not know |  |  |
|  |  |  |  |  |  |  |
| **A’ = Oral health related to quality of life/well-being** | **Q5** | **Do you feel other children make fun of you because of your teeth?** | | | | |
|  |  |  | **1** | Yes |  |  |
|  |  |  | **2** | No |  |  |
|  |  |  | **99** | Not sure/Do not know |  |  |
|  |  | **Q5.1** | **[if yes] - How do you feel about it? Using faces logo** | | | |
|  |  |  | **1** |    \| Okay \| \| --- \| | | |
|  |  |  | **2** | Sad | | |
|  |  |  | **3** | Very Sad (Makes me cry) | | |
|  |  |  | **99** | I don't know / I'm not sure | | |
|  |  |  |  |  |  |  |
| **B = Behaviour change** -Toothbrushing timing and frequency | **Q6** | **Did you brush your teeth yesterday?** | | | | |
|  |  |  | **1** | Yes | | |
|  |  |  | **2** | No | | |
|  |  |  | **99** | I can't remember / Not sure |  |  |
|  |  | **Q6.1** | **[if yes] - How many times?** | | | |
|  |  |  | **1** | 1 time |  |  |
|  |  |  | **2** | 2 times |  |  |
|  |  |  | **3** | 3 times |  |  |
|  |  | **Q6.2** | **Did you brush your teeth today?** | | | |
|  |  |  | **1** | Yes | | |
|  |  |  | **2** | No | | |
|  |  |  | **99** | Not Sure / can't remember |  |  |
|  |  | **Q6.2.1** | **[if yes] - How many times?** | | | |
|  |  |  | **1** | 1 time |  |  |
|  |  |  | **2** | 2 times |  |  |
|  |  |  | **3** | 3 times |  |  |
|  |  | **Q6.3** | **How often do you brush your teeth at home?** | | | |
|  |  |  | **0** | Not at home |  |  |
|  |  |  | **1** | 1 time |  |  |
|  |  |  | **2** | 2 times |  |  |
|  |  |  | **3** | 3 times |  |  |
|  |  | **Q6.4** | **Most days, do you brush your teeth both in the morning and in the evening?** | | | |
|  |  |  | **1** | Yes |  |  |
|  |  |  | **2** | No |  |  |
|  |  |  | **99** | Not sure |  |  |
|  |  | **Q6.5** | **When do you brush your teeth? (select all that apply)** | | | |
|  |  |  | **1** | Morning before breakfast |  |  |
|  |  |  | **2** | Morning after breakfast |  |  |
|  |  |  | **3** | After lunch |  |  |
|  |  |  | **4** | Evening but eat/drink after brushing | | |
|  |  |  | **5** | Evening and no eating/drinking after brushing | | |
|  |  |  | **6** | Other time of day |  |  |
|  |  |  | **99** | Do not know/ can't remember |  |  |
|  |  | **Q6.6** | **What do you use to brush your teeth? (Show example to illustrate if needed)** | | | |
|  |  |  | **1** | Toothbrush and toothpaste |  |  |
|  |  |  | **2** | Toothbrusonly |  |  |
|  |  |  | **3** | Chewing stick/Miswak |  |  |
|  |  |  | **4** | Charcoal |  |  |
|  |  |  | **5** | Other |  |  |
|  |  |  | **99** | Do not know |  |  |
|  |  | **Q6.7** | **Do you use fluoride toothpaste? (Show example of different example of toothpaste)** | | | |
|  |  |  | **1** | Yes |  |  |
|  |  |  | **2** | No |  |  |
|  |  |  | **99** | Do not know / can't remember |  |  |
|  |  |  |  |  |  |  |
| **A’ = Oral health related to quality of life/well-being KNOWLEDGE** | **Q7** | **Do you think it is important to brush your teeth every day?** | | | | |
|  |  |  | **1** | Yes |  |  |
|  |  |  | **2** | No |  |  |
|  |  |  | **0** | Not sure / don't know / can't remember |  |  |
|  |  | **Q7.1** | **[if yes] - How often should you brush your teeth every day?** | | | |
|  |  |  | **1** | Once |  |  |
|  |  |  | **2** | Twice |  |  |
|  |  |  | **3** | More than twice |  |  |
|  |  |  |  |  |  |  |
| **C’ = Socio-economic factors** -Absenteeism/missed days from school due to OH (reported by teachers) | **Q8** | **Number of missed days at school due to OH issues to be reported by teachers in liaison notebook since beginning of school year/calendar.** | | | | |
|  |  |  |  | [Input Field] | | |
|  |  |  |  |  |  |  |
