# Supplementary figures and images for: A 21-Day School-Based Toothbrushing Intervention in Children Aged 6 to 9 Years in Indonesia and Nigeria: Protocol for a Two-Arm Superiority Randomized Controlled Trial
Source: JMIR Res Protoc. 2020 Feb 21;9(2):e14156. doi: 10.2196/14156 (PMC7060496; doi:10.2196/14156)

**
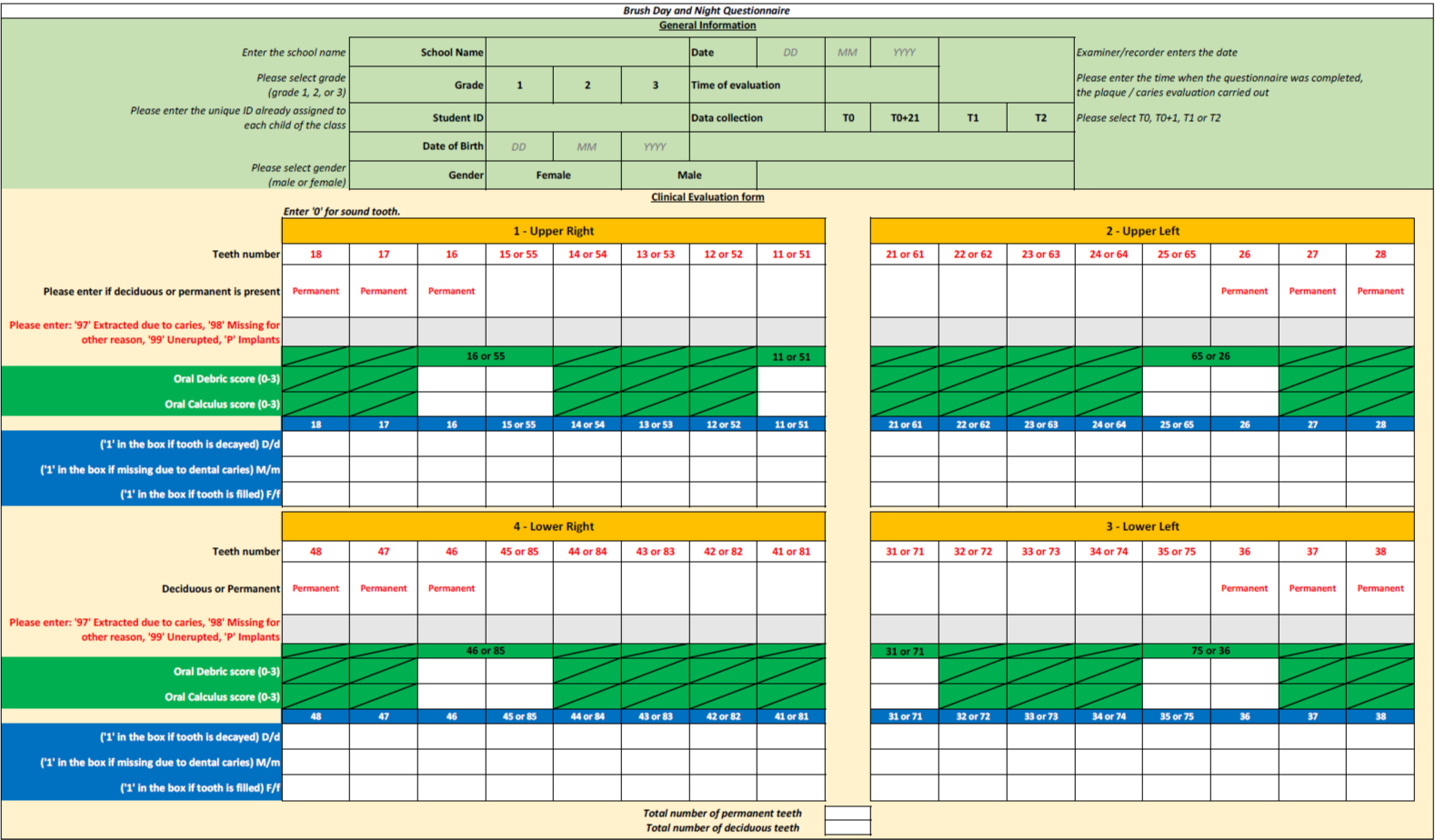
Multimedia Appendix 6. DMFT and OHIs paper form for data collection**

Supplement: Multimedia Appendix 6 [file resprot_v9i2e14156_app6.docx]
